# ERK5 Mediated Signalling in Diabetic Retinopathy 

**Published:** 2015

**Authors:** Yuexiu Wu, Subrata Chakrabarti

**Affiliations:** Department of Pathology, Schulich School of Medicine, Western University, London, Ontario, Canada

**Keywords:** Endothelial Cells, ERK5, Diabetic Retinopathy

## Abstract

Diabetic retinopathy is the lead among causes of blindness in North America. Glucose-induced endothelial injury is the most important cause of diabetic retinopathy and other vascular complications. Extracellular signal-regulated kinase 5 (ERK5), also known as big mitogen-activated protein kinase 1 (BMK1), is a member of mitogen-activated protein kinases (MAPK) family. Physiologically, it is critical for cardiovascular development and maintenance of the endothelial cell integrity. Extracellular signal-regulated kinase 5 is protective for endothelial cells under stimulation and stress. Decreased activation of ERK5 results in increased endothelial cell death. Extracellular signal-regulated kinase 5 signaling may be subject to alteration by hyperglycemia, while signaling pathway including ERK5 may be subject to alteration during pathogenesis of diabetic complications. In this review, the role of ERK5 in diabetic macro- and microvascular complications with a focus on diabetic retinopathy are summarized and discussed.

## INTRODUCTION

Chronic complications are the leading cause of mortality and morbidity in all types of diabetes (1, 2). Vascular endothelium is a primary organ affected in chronic diabetic complications wherein it acts both the target organ and potential mediator (1, 3). Chronic complications typically develop after 10 to 20 years of diabetes, and include both macroangiopathy and microangiopathy. Macroangiopathy is an accelerated form of atherosclerosis, a pathological process initiated by injury of endothelial cells seen in diabetes. This increases the risk of myocardial infarction, stroke, intermittent claudication and the ischemic gangrene (4).

Diabetes also causes microvascular complications such as diabetic retinopathy (DR) and nephropathy (5). Diabetic retinopathy is a severe complication of diabetes, manifesting primarily as vascular changes (structural and functional) in the retina. Diabetic retinopathy may result in vision loss, and it is the most common cause of blindness in North America in the age group 25–74 years (6). It has two phases, non-proliferative diabetic retinopathy (NPDR) and proliferative diabetic retinopathy (PDR) (7, 8). In NPDR phase, the vessels in the retina are weakened and leaky, forming microaneurysms and retinal hemorrhages, which leads to decreased vision. Proliferative diabetic retinopathy is an advanced stage in which new, but fragile, therefore delicate blood vessels develop on the surface of the retina or on the optic disk. Consequently, they rupture easily what makes the cause to tractional retinal detachment and blindness (9). Several growth and vasoactive factors are implicated in the development of PDR (10). Vascular endothelial growth factor (VEGF) plays a significant role in mediating intraocular neovascularization in patients with DR (11). Inhibition of ocular VEGF by intravitreal injection of anti-VEGF drug has emerged as a promising treatment for PDR (12, 13).

Diabetic nephropathy is a progressive kidney disease caused by microangiopathy in the renal glomeruli. It is characterized by nephrotic syndrome and diffuse glomerulosclerosis (14) and is a common cause of dialysis in Western countries.

## HYPERGLYCEMIA IS DIRECTLY RELATED TO ENDOTHELIAL DYSFUNCTION IN DIABETES

Diabetes-associated conditions such as hypertension, dyslipidemia and insulin resistance are correlated to impaired endothelial function (1, 2, 4). However, hyperglycemia is most commonly causally associated with endothelial dysfunction in chronic diabetic complications such as DR (1, 15). Evidences demonstrate impaired endothelial vasodilator function during either acute or chronic hyperglycemia both in human (16-18) and in animal diabetes (19, 20). In addition, hyperglycemia is known to increase endothelial permeability to macromolecules, delay cell replication, increase the secretion of sclerotic matrix proteins, increase adhesive properties for leukocytes and decrease the secretion of the pro-fibrinolytic agents, such as tissue plasminogen activator (tPA) (1). Both the Diabetes Control and Complications Trial (DCCT) and the United Kingdom Prospective Diabetes Study (UKPDS) have demonstrated correlations between poor glycemic control and increased incidences of microvascular complications in patients with diabetes (21, 22). Other clinical trials have also shown that macrovascular complications such as coronary (23) and peripheral artery disease (24) are related to glycemic levels.

## STRUCTURE OF ERK5

**Figure 1:**
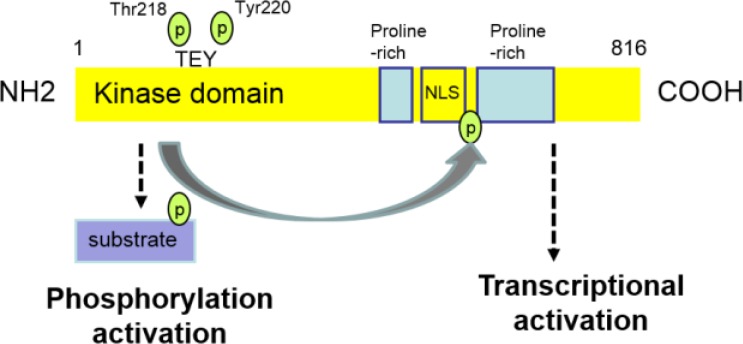
Structure and activation of ERK5.

Human ERK5 is 816 amino acids protein of with a predicted molecular mass of 98 kDa. Extracellular signal-regulated kinase 5 is encoded by MAPK7 gene, present in the majority of mammals (sharing 80-98% homology). It is more than twice the size of the other MAPKs due to its unique C-terminal. The N-terminal of MAPK’s catalytic domain share more than 50% homology with ERK1/2, which contains the Thr–Glu–Tyr (TEY) dual phosphorylation pattern in the activation loop (Fig. 1) (29). The C-terminal of ERK5 contains a nuclear localization signal (NLS) crucial for the nuclear localization of ERK5 upon stimulation; and two proline-rich regions that may serve as binding sites for Src homology 3 (SH3) domain containing proteins (29,32,33) (Fig. 1).

## KINASE ACTIVATION OF ERK5

**Figure 2:**
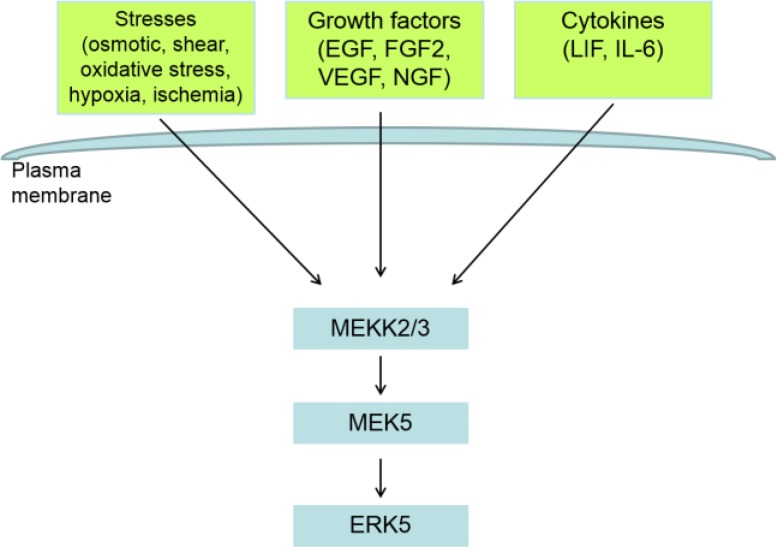
Activators of ERK5 Pathway.

Mitogen- activated protein kinases signaling cascade consists of three sequentially activated kinases: MEKK, MEK, and MAPK. These kinase module relay signals from extracellular agonists to cellular targets. The signaling modules in the ERK5 pathway are composed of MEKK2/MEKK3, MEK5 and ERK5 (Fig. 2) (28, 29, 38, 39). MEKK2/MEKK3 phosphorylate MEK5 on Ser311 and Thr315, resulting in an increase in MEK5 activities (38). Extracellular-signal-regulated kinase 5 is activated by dual phosphorylation at Thr218/Tyr220 by an upstream kinase MEK5 (28, 29, 40). MEK5 preferentially phosphorylates ERK5 on Thr218, which might induce a conformational change and subsequent phosphorylation of Tyr220 (41). Active ERK5 can undergo auto-phosphorylation on the C-terminal at a number of residues including Thr28, Ser421, Ser433, Ser496, Ser731, and Thr733, leading to an enhancement of ERK5 transcriptional activity as described below. Activated ERK5 also phosphorylates MEK5 at residues 129, 137, 142 and 149 which are located in the region that is thought to interact with ERK5 (41). PKCζ, an atypical protein kinase C, has been reported to interact with MEK5 in EGF-induced activation of ERK5 (42, 43). Interestingly, a recent study demonstrated that PKCζ is directly associated with ERK5. PKCζ mediates inhibitory phosphorylation of ERK5 by binding and phosphorylating serine 486, thus suppressing ERK5 function in TNFα-mediated inflammatory process (44).

The signaling modules in the ERK5 pathway are composed of MEKK2/MEKK3, MEK5, and ERK5. ERK5 is activated by a variety of stimuli. It can be activated by serum and a range of growth factors including EGF, FGF2, VEGF, and nerve growth factor (NGF). It can also be activated by cytokines such as leukemia inhibitory factor (LIF) and IL-6. Additionally, range of stress stimuli such as osmotic (58), fluid shear(30), or oxidative stresses; hypoxia (59) or ischemia (60) may activate ERK5.

G-proteins are involved in the activation of ERK5 by growth factors (61). In addition, studies have shown that PKCζ mediates ERK5 activation by G protein-coupled receptors (GPCR) (42, 44, 62). It has been also reported that G protein acts as an adaptor protein in PKCζ-mediated ERK5 activation by GPCR (62).

## TRANSCRIPTIONAL ACTIVATION OF ERK5

The C-terminal region of ERK5 contains a transcriptional activation domain, which is required for maximal transcriptional activity of target molecules (32, 45, 46). Activated ERK5 phosphorylates itself at the C-terminal at a number of residues (41) and auto-phosphorylation of C-terminal region of ERK5 leads to enhanced transcriptional activity (45, 47). Once stimulated, phosphorylation of ERK5 results in the activation of the kinase activity. Extracellular-signal-regulated kinase 5 phosphorylates both downstream target molecules and their C-terminal region (Fig. 1). Thus, auto-phosphorylation of the C-terminal leads to a further increase in the transcription activity of target molecules (47). In addition, Morimoto et al. showed that the activated kinase activity of ERK5 is required for the C-terminal mediated transcriptional activation of downstream targets. Mutation of phosphorylatable Thr and Ser residues to unphosphorylatable Ala significantly reduces the transcriptional activation effect of ERK5 (47). Interestingly, C-terminal also regulates the kinase activation of N-terminal. Deletion of C-terminal results in a dramatic increase in kinase activation of N-terminal (32).

## REGULATORS OF ERK5 SIGNALING

Similar to other MAPKs, ERK5 is activated by a variety of stimuli (Fig. 2). Studies have revealed that it is activated by serum (48), a range of growth factors including epidermal growth factor (EGF) (49), fibroblast growth factor-2 (FGF-2) (50), VEGF (31), and by cytokines such as LIF (51) and interleukin 6 (IL-6) (52). Additionally, NGF, use the ERK5 pathway to mediate its effects on neuronal cells, ECs as well as other cell types (53-56). We found that recombinant NGF stimulated ERK5 activation in the basal and high glucose conditions in ECs (57).

## SUBSTRATES OF ERK5 SIGNALING

A number of molecules have been identified as substrates of the ERK5 pathway. The transcription factors of the myocyte enhancer factor 2 (MEF2) family are best-characterized substrates of ERK5 (48, 63, 64). MEF2 is a four-membered family of transcription factors including MEF2A, MEF2B, MEF2C, and MEF2D. ERK5 phosphorylates and activates MEF2A, MEF2C and MEF2D, but not MEF2B (48, 63). The C-terminal tail of ERK5 contains an MEF2-interacting region and a transcriptional activation domain essential for co-activation of MEF2 (45). Activation of the MEF2 by the ERK5 is indispensable for EC survival and proliferation (48, 65). In addition, Krueppel-like factor 2 (KLF2) is identified as an ERK5 responsive gene and ERK5 drives KLF2 transcription by activating MEF2 (66). Krueppel-like factor 2 plays an important role in regulating inflammation, angiogenesis and maintaining the vascular quiescence (66-70). Studies in our lab suggest that MEF2 and KLF2 may be mediators of ERK5 signaling in the regulation of vasoactive factors involved in chronic diabetic complications (36, 37, 57). It has been shown that KLF2 lentivirus transfection inhibits transforming growth factor beta 1 (TGFβ1) signaling (71). We found a significant inhibition of TGFβ1 signaling after CAMEK5 transfection, and an increase of TGFβ1 mRNA after siERK5 transfection, suggesting that TGFβ1 signaling mediates the effect of ERK5 in high glucose conditions (57).

Ets-domain transcription factor (Sap1a) as well as serum- and glucocorticoid-inducible kinase (SGK) have also been identified as the downstream targets of ERK5 and play an important role in cell proliferation induced by growth factors (31, 55). Moreover, the ERK5 signaling pathway stimulates the transcriptional activity of c-Fos and Fra-1 (fos-related antigen 1) and members of the AP-1 (activator protein 1) family (46). Other downstream substrates of ERK5 include Cx43 (connexin 43 - a gap junction protein) (72), BAD (Bcl2 associated death promoted - a pro-apoptotic member of Bcl-2 family) (73), C-Myc proto-oncogene (74) and CREB (cAMP response element binding protein) (54).

## ERK5 IN ENDOTHELIAL CELLS

Extracellular-signal-regulated kinase 5 is highly expressed in the ECs and is essential for maintaining endothelial function and blood vessel integrity (31). Extracellular-signal-regulated kinase 5-deletion is lethal as seen in ERK5-/- mice who die around embryonic day 10 due to cardiovascular defects (59, 75, 76). Similar phenotypic abnormalities are observed in the MEKK3−/−, MEK5−/− and MEF2−/− embryos, suggesting that the MEKK3/MEK5/ERK5/MEF2 cascade is critical to the cardiovascular development (77-79). Additional studies employing targeted deletion of ERK5 gene in mice have shown that ERK5 is essential in EC physiology, but not in the cardiac development (80). Endothelial cells specific ERK5 ablation generates the same heart defects as those observed in global ERK5 knockout mutants, whereas cardiomyocyte specific ERK5 deletion mice are normal (80). These results indicate that ERK5 is critical for endothelial cell function and that the abnormal heart development in the mice lacking ERK5 is a consequence of endothelial cell dysfunction (80). Additionally, ERK5 is required to maintain vascular integrity in adult mice. Adult mice display hemorrhages in multiple organs and die within 2–4 weeks after deletion of ERK5 (80). In addition to these in vivo studies, ERK5 has been shown to be essential for endothelial cells survival in vitro (73, 80). Deletion of ERK5 induces profound endothelial cell apoptosis. Introduction of exogenous ERK5 can prevent endothelial cells from cell death (80). Similarly, activation of ERK5 by constitutively active MEK5 (CAMEK5) significantly improved cell viability and decreased apoptosis induced by growth factor deprivation (73). In addition, CAMEK5 inhibited growth factor deprivation-induced apoptosis, whereas dominant negative ERK5 (DNERK5) stimulated apoptosis in endothelial cells (73). ERK5 pathway also mediates the shear stress-induced antiapoptotic effect in endothelial cells (30, 73). Inhibition of ERK5 activity by overexpression of dominant negative ERK5 reduces endothelial-protective effect of shear stress (73). Analysis of antiapoptotic mechanisms of ERK5 showed that MEF2C, a direct substrate of ERK5 mediates endothelial cell survival signal (80).

## ERK5 IN DIABETIC RETINOPATHY

Our study has demonstrated the existence of the initial ERK5 activation in ECs because of glucose administration, followed by decreased activation upon prolonged glucose exposure. Decreased ERK5 signaling may contribute to increased vasoactive factors and extracellular matrix accumulation (36, 37, 57). In keeping with our data, a previous study showed glucose-induced initial ERK5 activation in pulmonary artery ECs (81).

Endothelin-1 (ET-1) is a potent vasoconstriction factor whose role has been implicated in the pathogenesis of DR (82-84). Blockade ET increases retinal blood flow and prevents DR (82, 83). Decreased ERK5 activation and increased ET-1 expression were observed in ECs treated with high glucose (36). We also observed similar changes in retinal tissues of diabetic rats (36). Activation of ERK5 by CAMEK5 upregulated KLF2 and suppressed both basal and glucose-induced ET-1 expression in ECs. In contrast, ERK5 siRNA transfection resulted in decreased ERK5, KLF2 and increased ET-1 expression (36).

Vascular endothelial growth factor is a major contributor of retinal neovascularization in DR (85, 86). Elevated VEGF mRNA and protein expression have been confirmed in the patient with DR (87-89). Extracellular-signal-regulated kinase 5 has been shown to take part in the regulation of VEGF. Vascular endothelial growth factor expression is upregulated in ERK5 knockout mice (59, 66, 90, 91). Further in vitro studies showed that ERK5 repressed VEGF expression by negatively regulating hypoxia inducible factor-1α (HIF1α) in bovine lung microvascular ECs (92). Hypoxia inducible factor-1α is a strong mediator of angiogenesis in hypoxia by regulating VEGF (93, 94). High glucose induces a state of pseudo-hypoxia in diabetic complications (95, 96). It is, therefore, possible that decreased ERK5 signaling may promote glucose-induced VEGF production and angiogenesis via HIF1α. A recent study has further shown that constitutive activation of ERK5 signaling strongly inhibited EC migration, whereas ERK5 siRNA transfection increases migration (97). Similarly, our experiments showed that ERK5 siRNA enhances tube formation and VEGF expression in the ECs. Constitutively activation of ERK5 by CAMEK5 reduced both basal and glucose-induced VEGF expression (37). In addition, we observed decreased ERK5 signaling and increased VEGF expression in the retina of diabetic rats (37).

**Figure 3:**
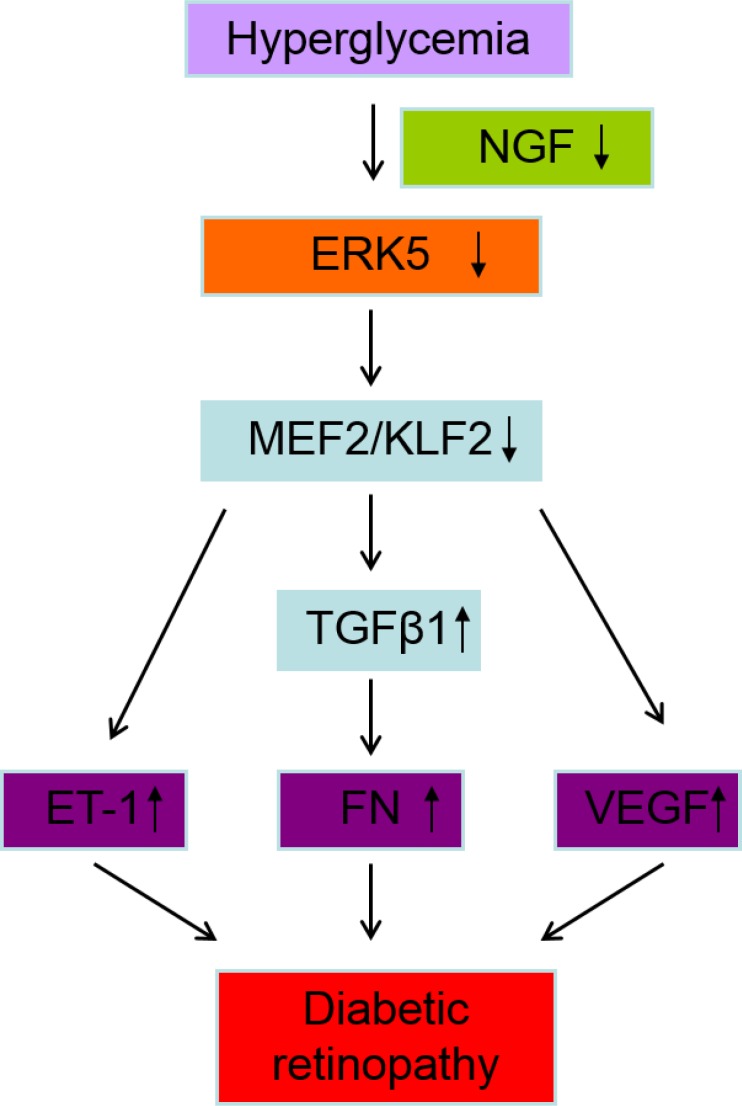
A diagrammatic representation of the main conclusions of this study, outlining possible role of ERK5 in DR.

Fibronectin (FN) is an important component of the extracellular matrix, which plays a significant role in EC adhesion, migration, growth and proliferation (98, 99). FN overproduction is a characteristic feature of DR. Studies in our lab, and others have shown that the synthesis of FN is upregulated in diabetes and ECs treated with glucose (100-103). We have found a significant decrease of FN mRNA and protein following CAMEK5 transduction in basal and high glucose conditions (57). In contrast, ERK5 siRNA transfection and DNMEK5 transduction lead to an increase of FN synthesis. Moreover, our study has demonstrated that TGFβ1 signaling mediates the effect of ERK5 on FN. Furthermore, we have observed that FN expression in retinal tissues of diabetic rats is increased while ERK5 activation is decreased (57). These data suggested that decreased ERK5 signaling is important in glucose-induced FN overproduction and DR. A diagrammatic representation of such mechanisms is outlined in Fig. 3.

Hyperglycemia decreased activation of ERK5, which lead to upregulation of ET-1, VEGF, FN expression, and function, subsequently possibly contributing to DR. NGF mediated hyperglycemia-induced ERK5 alteration. ERK5 exerted its effect on endothelial cells via MEF2/KLF2 and TGFβ1.

## ERK5 IN OTHER DIABETIC VASCULAR COMPLICATIONS

Macroangiopathy in diabetes is mainly due to an accelerated form of atherosclerosis (4). Steady and laminar blood flow is known to be atheroprotective and has been shown to be a strong activator of ERK5 (30). Also, ERK5 activation has been demonstrated to be atheroprotective. Increased plaque formation is observed in inducible EC-specific ERK5 knockout mice (104). In addition, inhibition of ERK5 activity by dominant negative ERK5 reduces the endothelial cell-protective effect of shear stress (73), indicating that the ERK5 mediates the shear stress-induced antiapoptotic effect in endothelial cells. This may be mediated by phosphorylation of BAD (73). Sohn et al. revealed that KLF2 mediates endothelial-protective effect of ERK5 (66). KLF2 is a critical transcriptional regulator for the vasoprotective effect of shear stress (67,105). In addition, laminar flow-induced ERK5 activation has been shown to confer an atheroprotective effect by inducing peroxisome proliferator-activated receptor gamma (PPARγ) (106) and inhibiting tumor necrosis factor α (TNFα) mediated adhesion molecule expression in endothelial cells (107).

However, SUMOylation inhibits a protective effect of ERK5 in diabetes (108), as small ubiquitin-like modifier (SUMO) covalently attaches to certain residues of specific target proteins and negatively regulates transcription factors (109,110). Increased ERK5 SUMOylation in diabetes inhibits shear stress-mediated ERK5’s transcription activity. Subsequently decreased KLF2 and endothelial nitric oxide synthase (eNOS) expression lead to endothelial dysfunction and accelerated atherosclerosis in diabetes (108). Extracellular-signal-regulated kinase 5 activity is also suppressed by p90 ribosomal S6 kinase (p90RSK) which is found to be increased in diabetic mouse vessels. p90 ribosomal S6 kinase -mediated reduction of ERK5 activity increased adhesion molecule1 and reduced eNOS expression, which contribute to atherosclerosis in diabetes (104).

Some studies have been performed to investigate further the role of ERK5 on diabetic nephropathy. A recent study on renal epithelial cells showed that the overexpression of ERK5 provided protection against renal ischemia-reperfusion injury (111). However, studies in mesangial cells have contradictory results. It has been reported that ERK5 activation stimulates mesangial cell proliferation and extracellular matrix accumulation (112,113). Similarly, ERK5 increases mesangial cell viability and collagen matrix accumulation in glomerulonephritis (114). The differences between mesangial cells and renal epithelial cells indicate that ERK5 signaling may regulate extracellular matrix production in a cell type-specific manner.

## CONCLUSION

Chronic vascular complications are leading causes of morbidity and mortality in diabetes. Extracellular-signal-regulated kinase 5signaling plays a significant role in maintaining vascular integrity. A number of studies demonstrated that ERK5 is protective against endothelial injury in high glucose concentrations, and it exerts its effects by acting on multiple factors that are involved in regulating endothelial function. Hence, ERK5 may be a potential target for prevention and treatment of DR and other chronic diabetic complications.
